# Social Disparities in Mental Health Service Use Among Children and Youth in Ontario: Evidence From a General, Population-Based Survey

**DOI:** 10.1177/07067437221144630

**Published:** 2022-12-12

**Authors:** Mahdis Kamali, Jordan Edwards, Laura N. Anderson, Eric Duku, Katholiki Georgiades

**Affiliations:** 1 Department of Health Research Methods, Evidence and Impact, 3710 McMaster University, Hamilton, Ontario, Canada; 2 204242 Offord Centre for Child Studies, Ron Joyce Children's Health Centre, Hamilton, ON, Canada; 3 Department of Psychiatry and Behavioural Neurosciences, 3710 McMaster University, Hamilton, Ontario, Canada

**Keywords:** immigrant health, mental health disparities, service utilization, children and youth, mental healthcare need

## Abstract

**Objectives:**

To examine differences in mental health-related service contacts between immigrant, refugee, racial and ethnic minoritized children and youth, and the extent to which social, and economic characteristics account for group differences.

**Methods:**

The sample for analyses includes 10,441 children and youth aged 4–17 years participating in the 2014 Ontario Child Health Study. The primary caregiver completed assessments of their child's mental health symptoms, perceptions of need for professional help, mental health-related service contacts, experiences of discrimination and sociodemographic and economic characteristics.

**Results:**

Adjusting for mental health symptoms and perceptions of need for professional help, children and youth from immigrant, refugee and racial and ethnic minoritized backgrounds were less likely to have mental health-related service contacts (adjusted odds ratios ranged from 0.54 to 0.79), compared to their non-immigrant peers and those who identified as White. Group differences generally remained the same or widened after adjusting for social and economic characteristics. Large differences in levels of perceived need were evident across non-migrant and migrant children and youth.

**Conclusion:**

Lower estimates of mental health-related service contacts among immigrant, refugee and racial and ethnic minoritized children and youth underscore the importance and urgency of addressing barriers to recognition and treatment of mental ill-health among children and youth from minoritized backgrounds.

## Résumé

**Objectifs:**

Examiner les différences des contacts avec les services de santé mentale entre les immigrants, les réfugiés, les enfants et les jeunes minorisés d’origine raciale et ethnique, et la mesure dans laquelle les caractéristiques sociales et économiques représentent les différences des groupes.

**Méthodes:**

L’échantillon servant aux analyses comprend 10 441 enfants et jeunes de 4 à 17 ans participant à l’Étude sur la santé des enfants de l'Ontario de 2014. Le soignant principal a procédé à des évaluations des symptômes de santé mentale de son enfant, des perceptions du besoin d’aide professionnelle, des contacts avec les services de santé mentale, des expériences de discrimination et des caractéristiques sociodémographiques et économiques.

**Résultats:**

Après ajustement pour des symptômes de santé mentale et des perceptions du besoin d’aide professionnelle, les enfants et les jeunes de milieux immigrants, réfugiés et de milieux minorisés raciaux et réfugiés étaient moins portés à avoir des contacts avec les services de santé mentale (les RCa variaient entre 0.54–0.79), comparativement à leurs pairs non-immigrants et à ceux qui s’identifiaient comme Blancs. Les différences de groupes demeuraient généralement les mêmes ou grandissaient après ajustement pour les caractéristiques sociales et économiques. De grandes différences des niveaux du besoin perçu étaient évidentes chez les enfants et les jeunes migrants et non-migrants.

**Conclusion:**

Les faibles estimations des contacts avec les services de santé mentale chez les enfants et les jeunes immigrants, réfugiés, et de minorité raciale et ethnique soulignent l’importance et l’urgence de s’occuper des obstacles à la reconnaissance et au traitement de la santé mentale chez les enfants et les jeunes de milieux minorisés.

## Introduction

In Ontario, it is estimated that 18% to 22% of children and youth aged 4 to 17 years will have a mental disorder, at any given time, associated with substantial distress and impairment.^1^ Less than one-third of these children and youth will have contact with a specialized mental healthcare provider.^1^ These mental health treatment gaps highlight the need to identify population subgroups less likely to receive mental healthcare and address barriers to recognition and treatment.^2^

Evidence from Canada and the United States designed to identify factors associated with disparities in mental health service use among youth point to a variety of sociodemographic and family factors including gender, migrant status, racial and ethnic minoritized background, poverty, parental education, and family structure.^1,3–14^ Lower levels of service use for mental healthcare have been consistently documented for females, children living in poverty and with lower parental education levels, families with 2 biological parents and youth from immigrant and racial and ethnic minoritized backgrounds.^1,3–14^

In Canada, children and youth from immigrant and refugee (hereafter referred to as migrant) backgrounds are much less likely to receive mental healthcare, compared to non-migrant peers.^1,13,14^ Evidence syntheses and systematic reviews follow the same trend: children and youth from migrant families are much less likely to access mental health services compared to individuals native to the country being examined.^15,16^ Migration can often fuel health inequities by exposing families to economic decline, limited access to healthcare and education, and poor living and work conditions.^17–19^ Even within migrant subgroups differences in mental health have been documented, with refugees exhibiting increased mental health symptom severity and hospitalization.^6,20–22^ Refugees are distinct from immigrants and should be examined in this way as their resettlement in Canada is considered involuntary and a result of fear of persecution, war or armed conflict.^23^

Andersen's behavioural model fits within the paradigm of variance in service use by conceptualizing the determinants into 3 broad categories.^24^ It posits that the causal pathway to the use of health services begins with predisposing characteristics (i.e., demographic factors, health beliefs), then personal and community enabling factors (i.e., family resources, use of social supports) and lastly, the need for health services (evaluated and perceived need).^24^ In the context of children's pathway to mental health services, parents are conceptually identified as facilitating the initiating step to seek service through their ability to perceive the need for help for emotional and behavioural symptoms in their children.^25,26^ To comprehensively conceptualize need for mental health services, it is important to account for mental health symptoms (evaluated need), as well as a parent-reported perceived need for services. It can be maintained that social context plays an important role in perceptions.^27^ For instance, perceived discrimination has often been cited as an important social contextual stressor accounting for differences in mental health disparities among racial and ethnic minoritized populations.^28–30^ However, its influence on mental health service utilization within migrant groups is lacking. We hypothesize that predisposing factors (gender, migrant status, racial and ethnic background), personal/community enabling resources (family structure, household income, social assistance, parental education and employment, exposure to discrimination) and need for services (mental health symptoms and perception of need) represent important correlates of mental health help-seeking behaviours among children, youth and their families in Ontario.

While evidence shows that the use of specialized mental healthcare among children and youth with mental disorders is low, the factors contributing to these disparities, particularly among immigrant, refugee, racial and ethnic minoritized children and youth in a Canadian context remain largely unknown.^1,4,22^ Moreover, our understanding is primarily based on health administrative data, which is limited to physician-based contacts, including outpatient and inpatient settings. Therein, we are unable to account for the level of mental health need, as well as services provided outside of hospitalization and physician-based services. Evidence documenting patterns of mental health service use across sectors and settings clearly demonstrates that youth frequently utilize services outside of the healthcare system.^1,7,8,31,32^ Sole reliance on administrative data results in a significant proportion of mental health-related service contacts being missed, while also restricting our evidence to a subset of adolescents who received care from a mental health professional for mental health-related concerns. Identifying correlates of mental health service-related contacts and quantifying disparities requires a comprehensive approach that incorporates all relevant settings and sectors that deliver mental healthcare to children and youth and those who have, and have not, accessed care. Most available evidence on the sector and setting specific correlates of mental healthcare has been derived from the United States and Australia, with limited applicability to a Canadian context due to variations in healthcare systems, financing and policies.^7,8,31,32^

The present study uses general population data to examine correlates of mental health-related service contacts among children and youth aged 4 to 17 years in Ontario.^33^ Specifically, the study objectives are to (1) quantify differences in mental health-related service contacts between immigrant, refugee, racial and ethnic minoritized children and youth relative to non-migrants; and (2) examine the extent to which social and economic characteristics account for group differences. With Canada's changing population landscape, it is necessary to understand not only the magnitude of mental healthcare disparities in migrant subgroups, but also the social determinants contributing to them to achieve equitable access to mental healthcare in Ontario.

## Methods

Ethics approval for all study procedures was obtained through the Hamilton Integrated Research Ethics Committee at McMaster University (Hamilton, ON) and final ethics was obtained from the Research Ethics Board. The data are stored and accessible in Canada through the Statistics Canada Research Data Centres program.

### Study Design and Data Source

Data for analyses come from the 2014 Ontario Child Health Study (OCHS). The OCHS is a general population provincial epidemiologic study of child and youth health and mental disorder. Sampling for the OCHS was based on a 3-stage stratified, random cluster design using the 2014 Canada Child Tax Benefit (CCTB) file as a sampling frame to identify households with children aged 4 to 17 years. Data were collected from the person most knowledgeable (PMK) about the household and child (98.6% identified as the parent of a selected child). Between October 2014 and September 2015, a total of 10,802 children and youth aged 4 to 17 years nested in 6,537 families and 484 neighbourhoods (50.8% response) agreed to participate. Additional information on survey design, content, and data collection is available elsewhere.^34,35^

### Measures

*Mental health service contact*. The outcome of interest was the parent report of whether the child had a service contact regarding mental health concerns in the past 6 months. Parents were asked if they or their child saw or spoke to general healthcare providers (family doctor, pediatrician, and other health professionals), mental health providers (psychiatrist, psychologist, social worker, and other types of counsellors), school guidance counsellor or teacher/other adults at school, a combination of complementary/alternative medicine providers (religious or spiritual leader, alternative healers such as a naturopath or herbalist) or phone helpline or crisis hotline regarding their child's mental health in the past 6 months. Parents were also asked whether their child had gone to any of the following settings for mental health concerns: walk-in clinic, urgent care clinic or hospital, or an agency that provides mental health or addiction services for youth. A binary indicator of *any* service contact was derived indicating either no service contact or contact with at least 1 provider or in at least 1 service setting for mental health concerns (see Supplemental Appendix 1 for more details on variable definitions and coding). To examine the distribution of where children and youth are seeking care and from whom, service setting/sector and type of professional were classified into the following categories, as defined in Georgiades et al.^1^ (1) school (school-based setting), (2) specialized mental health (psychiatrist, psychologist, therapist, and mental health or addictions agency), (3) general healthcare (family doctor/pediatrician, walk-in clinic, urgent care, hospital emergency room), and (4) other (hotline, spiritual leader, and alternative health practitioner).

*Migrant status*. The migrant group was based on parent-reported country of birth and migration class. Respondents were classified as (1) immigrant, at least 1 parent arrived in Canada as a landed immigrant, (2) refugee, at least 1 parent came to Canada as a refugee, and (3) non-migrant, both parents were born in Canada.

*Sociodemographic factors.* Sociodemographic variables assessed in the OCHS are based on harmonized content developed by Statistics Canada. They include age (in years), sex (male = 0), parent racial and ethnic background (White = 0), Asian (East Asian, Southeast Asian, and South Asian), West Asian (West Asian, Arab), Black (Black African, Black Caribbean, Black Canadian or American), Other (Aboriginal, First Nations, Métis, Inuit, Latin American, Other), family structure (2 biological parents = 0), 2 parents (either 1 biological and 1 step-parent, adoptive parent, or foster parent; or 2 step-parents/adoptive/foster), single parent), household income below the low-income measure (0 = above low income),^36^ receipt of social assistance (no = 0), parental education level (bachelor's degree or higher = 0, diploma or trade certificate, high school degree or less) and employment (full-time = 0, part-time, looking, unemployed), urban–rural residency (rural = 0).^37^

*Internalizing and externalizing symptoms*. Internalizing and externalizing symptoms were measured through dimensional ratings from the 2014 OCHS-Emotional Behavioural Scales (OCHS-EBS).^38^ It was administered to the PMK describing their child's feelings and behaviours in the past 6 months rated on a 3-point Likert scale. Internalizing behaviours include 27 items measuring the frequency of symptoms of a generalized anxiety disorder (6 items), separation anxiety disorder (7 items), major depressive disorder (9 items), and social phobia/social anxiety disorder (5 items). Externalizing behaviours are derived from items measuring the frequency of symptoms of attention deficit hyperactivity disorder (8 items), oppositional defiant disorder (6 items), and conduct disorder (11 items). The OCHS-EBS has undergone psychometric evaluation and the reliability and validity of the scale have been well established.^38^ Subscale scores are calculated by summing together the coded responses across items within each disorder to produce a summative score. A higher score is indicative of greater symptom severity.

*Perception of need*. Parents were asked whether they felt their child needed professional help for emotional and/or behavioural problems (no = 0).

*Experienced discrimination*. Parents were asked to indicate whether they experienced discrimination or have been treated unfairly by others in Canada because of their ethnicity, culture, race, skin colour, language, accent, or religion (no = 0).

### Statistical Analysis

Children and youth aged 4 to 17 years old were eligible for inclusion in the analysis (*n* = 10,802). Restricting the sample to complete data across study variables resulted in a 3.3% reduction in the study sample (*n* = 10,441). Comparison between complete cases and those with missing data on all study variables showed no statistically significant differences.

Migrant group differences are presented across sociodemographic variables, mental health-related service contact and mental health symptoms and need. Cross-tabulations were used to calculate the prevalence of service contact by service setting/sector/professional and migrant status. Group differences were examined using analysis of variances (ANOVAs) and chi-square tests, as appropriate.

Sequential logistic regression analyses were undertaken to estimate odds ratios (ORs) and 95% confidence intervals (CIs) for associations between mental health service contact and study variables and to understand the extent to which these variables account for group differences. To examine associations between sociodemographic factors and mental health symptoms on service contact, Model 1 included youth demographic characteristics (age and sex), mental health symptoms and need (internalizing and externalizing symptoms, perception of need), migrant status and racial and ethnic background. In Model 2, family socio-economic and demographic characteristics were added: family structure, low income, receipt of social assistance, rural–urban residence, parental education and parental employment. Experienced discrimination was added in Model 3.

Due to the complex sample design of the 2014 OCHS, a total of 1,000 mean bootstrap weights generated by Statistics Canada were applied with an adjustment factor of 5 to obtain accurate standard errors.^35^ Sampling weights were also applied to all analyses to generate estimates that are representative of the target population of children and youth in Ontario. All analyses were conducted in the STATA statistical software system package version 14.0 (StataCorp., College Station, TX, USA).^39^

## Results

The distribution of sociodemographic characteristics and mental healthcare needs by migrant status are presented in Table 1. Significant group differences are observed for all study variables, except for age and sex. Over half of the study population are children from non-immigrant families (*n* = 5,974, 57.4%), immigrant children represented 37.3% of the sample, while refugee children and youth represented 5.5% of the total study sample. White children and youth comprised the largest group (62.6%), with immigrants (45.6%) and refugees (42.3%) predominately identifying as East, Southeast or South Asian. Despite immigrant and refugee parents exhibiting higher education levels, a significantly higher proportion were low income (22.4% of immigrant and 43.3% of refugee families, compared to 14.0% of non-immigrant families). Compared to non-immigrants, immigrant and refugee families were twice as likely to experience discrimination (24.7% in immigrants, 22.2% in refugees compared to 10.0% in non-immigrant families). Non-immigrant children and youth had higher levels of parent-reported mental health symptoms. Internalizing symptoms were significantly higher in non-immigrants compared to immigrant children and youth (Cohen's *d* = 0.13) and compared to refugees (*d* = 0.17). Similar patterns are observed in externalizing symptoms with non-immigrant children and youth exhibiting higher levels than immigrant (*d* = 0.20) and refugee (*d* = 0.22) children and youth. The prevalence of perceived need for help for emotional and behavioural concerns was also higher among non-immigrants (15.2%), compared to immigrants (5.9%) and refugees (5.6%).

**Table 1.** Weighted Characteristics of Sample by Migrant Status, Percentage and SE Unless Otherwise Stated.

Factor	Entire Sample (*n* = 10,441)	Migrant Status		
		Non-immigrant (*n* = 5,974)	Immigrant (*n* = 3,894)	Refugee (*n* = 573)
***Sociodemographic and economic***				
Child age (mean, SE)	10.6 (0.07)	10. 5 (0.09)	10.6 (0.12)	10.9 (0.32)
Child sex (% female)	48.7 (0.08)	48.9 (0.11)	47.9 (0.14)	48.6 (0.37)
Race/ethnicity				
White	62.6 (0.8)	89.9 (0.7)	27.6 (1.3)	11.5 (1.8)
East, Southeast, and South Asian	20.3 (0.7)	1.99 (0.36)	45.6 (1.5)	42.3 (3.8)
West Asian, Arab	31.7 (0.23)	0.19 (0.08)	7.31 (0.5)	7.39 (1.5)
Black	5.3 (0.34)	1.4 (0.24)	8.8 (0.70)	22.6 (3.2)
Other	8.6 (0.49)	6.6 (0.59)	10.6 (0.87)	16.1 (2.6)
Family structure				
Two biological parents	71.6 (0.78)	65.9 (1.1)	80.6 (1.05)	79.4 (2.9)
Two parents (2 step/foster/adoptive, 1 biological and 1 step/foster/adoptive)	8.33 (0.51)	11.5 (0.75)	4.6 (0.73)	1.59 (0.57)
Single parent	20.0 (0.68)	22.6 (0.99)	14.4 (0.83)	18.9 (2.8)
Household income (% below poverty line)	18.8 (0.53)	14.0 (0.62)	22.4 (0.95)	43.3 (3.5)
Social assistance	4.59 (0.26)	4.87 (0.37)	3.40 (0.36)	9.24 (1.39)
Parental education				
High school or less	12.5 (0.53)	12.4 (0.71)	10.7 (0.82)	22.6 (2.7)
Diploma or trade certificate	41.6 (0.88)	49.1 (1.2)	30.7 (1.4)	36.5 (3.8)
Bachelors or above	45.9 (0.87)	37.5 (1.1)	57.6 (1.4)	40.5 (3.7)
Parental employment				
Full-time	62.5 (0.82)	64.0 (1.1)	61.3 (1.3)	56.9 (3.5)
Part-time	15.7 (0.64)	15.4 (0.87)	16.8 (1.1)	12.0 (1.8)
Looking	10.9 (0.46)	10.0 (0.59)	11.4 (0.75)	16.2 (2.3)
Unemployed	10.8 (0.47)	10.6 (0.65)	10.3 (0.71)	14.7 (2.0)
Rural	12.9 (0.57)	20.4 (0.91)	3.95 (0.52)	0.96 (0.43)
Discrimination (% yes)	16.1 (0.64)	10.0 (0.67)	24.7 (1.3)	22.2 (2.9)
***Mental health care need***				
Internalizing symptoms (mean, SE)	5.75 (0.11)	6.40 (0.16)	4.91 (0.18)	4.66 (0.33)
Externalizing symptoms (mean, SE)	4.52 (0.88)	5.29 (0.13)	3.55 (0.12)	3.42 (0.28)
Perceived need for mental health professional help	11.2 (0.55)	15.2 (0.83)	5.9 (0.67)	5.6 (0.18)
***Mental health-related service contact***				
Any contact	25.3 (0.74)	32.4 (1.08)	15.9 (0.99)	14.7 (2.51)
Contact by setting:				
School	14.80 (0.61)	20.15 (0.94)	7.66 (0.71)	7.35 (1.84)
Specialized mental health	10.73 (0.53)	14.2 (0.80)	6.09 (0.66)	7.47 (2.19)
General healthcare	10.97 (0.53)	13.90 (0.80)	7.31 (0.72)	6.30 (1.72)
Complementary/alternative	3.00 (0.28)	4.30 (0.45)	—	—

All group differences statistically significant tests using analysis of variances (ANOVAs) and chi-square tests, as appropriate; —, does not meet the minimum unweighted cell count criteria for disclosure.

One-quarter (25.3%) of the sample made a contact with a professional for mental health concerns, with twice the number of non-immigrant children and youth making contact (32.4%) compared to their immigrant and refugee counterparts (15.9% and 14.7%, respectively). Schools were the most prevalent setting/sector for service contact among non-immigrant (20.1%) and immigrant (7.6%) children and youth, however, children from refugee families most often frequented specialized mental healthcare (7.5%).

Table 2 presents the adjusted ORs (aORs) and 95% CIs of the estimate of the association between study variables and mental health-related service contact in sequential models. Compared to non-immigrants, children and youth from immigrant (aOR: 0.63; 95% CI, 0.60 to 0.66) and refugee families (aOR: 0.64; 95% CI, 0.58 to 0.71) were less likely to have had a mental health-related service contact, after adjusting for mental health symptoms and need (Model 1). Similarly, children and youth from East, Southeast or South Asian families (aOR: 0.61; 95% CI, 0.57 to 0.65), West Asian and Arab families (aOR: 0.62; 95% CI, 0.56 to 0.70), Black families (aOR: 0.54; 95% CI, 0.50 to 0.58), and those who identified in the Other category (aOR: 0.79; 95% CI, 0.74 to 0.84) each had a reduced odds of mental health-related service contacts, relative to those who identified as White.

**Table 2.** Correlates of Mental Health-Related Service Contacts in the Past 6 Months, Adjusted Odds Ratios (aORs; 95% CI) (*n* = 10,441).

Factor	aOR (95% CI)		
	Model 1	Model 2	Model 3
Child age	0.97* (0.96 to 0.97)	0.96* (0.96 to 0.96)	0.96* (0.96 to 0.96)
Child sex (male)	1.02 (0.97 to 1.05)	1.04* (1.00 to 1.08)	1.04 (1.00 to 1.08)
Internalizing symptoms	1.06* (1.05 to 1.06)	1.06* (1.06 to 1.06)	1.06* (1.05 to 1.06)
Externalizing symptoms	1.04* (1.04 to 1.05)	1.04* (1.03 to 1.04)	1.04* (1.03 to 1.04)
Perception of need	5.77* (5.44 to 6.12)	5.45* (5.13 to 5.79)	5.45* (5.12 to 5.78)
Migrant status (ref non-immigrant)			
Immigrant	0.63* (0.60 to 0.66)	0.63* (0.60 to 0.66)	0.63* (0.59 to 0.66)
Refugee	0.64* (0.58 to 0.71)	0.64* (0.58 to 0.71)	0.64* (0.58 to 0.71)
Race/ethnicity (ref White)			
East, Southeast, and South Asian	0.61* (0.57 to 0.65)	0.59* (0.55 to 0.64)	0.59* (0.55 to 0.64)
West Asian, Arab	0.62* (0.56 to 0.70)	0.55*(0.49 to 0.62)	0.55* (0.49 to 0.62)
Black	0.54* (0.50 to 0.58)	0.47* (0.44 to 0.51)	0.47* (0.43 to 0.51)
Other	0.79* (0.74 to 0.84)	0.76* (0.71 to 0.82)	0.76* (0.71 to 0.82)
Family structure (ref 2 biological parents)			
Two parents (2 step/foster/adoptive, 1 biological and 1 step/foster/adoptive)		1.12* (1.04 to 1.20)	1.12* (1.05 to 1.20)
Single parent		1.32* (1.25 to 1.38)	1.31* (1.25 to 1.38)
Low income		1.14* (1.09 to 1.20)	1.14* (1.09 to 1.20)
Social assistance		1.83* (1.71 to 1.97)	1.83* (1.70 to 1.97)
Parental Education (ref Bachelors or above or higher)			
High school or less		0.69* (0.66 to 0.74)	0.69* (0.66 to 0.74)
Diploma, trade certificate		0.99 (0.96 to 1.03)	0.99 (0.96 to 1.03)
Parental Employment (ref full-time)			
Part-time		1.13* (1.08 to 1.19)	1.13* (1.08 to 1.19)
Looking		1.33* (1.26 to 1.41)	1.33* (1.26 to 1.41)
Unemployed		0.95 (0.91 to 1.00)	0.95 (0.91 to 1.00)
Rural (ref urban)		1.04 (0.98 to 1.10)	1.04 (0.98 to 1.10)
Discrimination			1.02 (0.97 to 1.07)

*p < .05.

Higher mental health need was associated with mental health-related service contact. Children with increased internalizing and externalizing symptoms were significantly more likely to make contact for mental health concerns (aOR: 1.06; 95% CI, 1.05 to 1.06; aOR: 1.04; 95% CI, 1.03 to 1.04, respectively). Perceived need for professional help for emotional and/or behavioural problems was the strongest predictor of mental health-related service contact (aOR: 5.77; 95% CI, 5.44 to 6.12).

In Model 2, family sociodemographic characteristics were added. Low income families and those who s received social assistance had increased odds of having a service contact for mental health concerns. Compared to families with 2 biological parents, children and youth living with either 2 adoptive parents or 1 biological parent and 1 adoptive/foster (aOR: 1.12; 95% CI, 1.04 to 1.20) and those living with a single parent had higher odds of mental health-related service contact (aOR:1.32; 95% CI, 1.25 to 1.38). The magnitude of the ORs associated with immigrant and refugee backgrounds remained relatively unchanged with the addition of sociodemographic variables in the sequential models. The final model included parental experiences of discrimination. After adjusting for all other study variables, the association between experiences of discrimination and mental health-related service contacts was not statistically significant (aOR: 1.02; 95% CI, 0.97 to 1.07).

## Discussion

This study enhances our understanding of the prevalence and determinants of mental health-related service contacts among children and youth in Ontario. We found higher levels of mental health symptoms and a perceived need for professional help for mental health concerns among non-immigrants, compared to immigrant children and youth. After adjusting for these group differences, our results highlight disparities in mental health-related service utilization among migrant groups, with immigrant children and youth less likely to seek services. A similar pattern was observed among racial and ethnic families, where they are almost 40% to 50% less likely to make contact compared to self-identified White families. Social and economic factors did not account for the observed disparities, pointing to the need to identify other underlying considerations that are contributing to differential help-seeking behaviours across underserved population subgroups.

Importantly, we found children and youth living in low income households, receiving social assistance, and having a single or no biological parent in the home were more likely to have mental health-related service contacts. The mechanisms contributing to these group differences are unclear, however, other studies with similar findings have prompted careful consideration of stressful social circumstances in the lives of vulnerable children and youth, and the importance of the family environment in forming mental health treatment patterns.^1,13,31,40^

Large group differences were documented in experiences of discrimination with nearly one-quarter of immigrant and refugee families reporting experiences of discrimination compared to one-tenth of non-immigrant families. While experiences of discrimination have been associated with mental health disparities, our findings suggest that parents who have experienced discrimination or unfair treatment were not less likely to access mental healthcare for their child.^29,30,41,42^ Caution is warranted when interpreting these results given the sole reliance on a single item to assess experiences of discrimination that were not specific to the healthcare context. It is also important to note that experiences of discrimination were added to our final analytic model which controlled for a number of sociodemographic and economic characteristics. Indeed, in unadjusted analyses reports of discrimination were associated with a reduced likelihood of mental health-related service contacts (OR: 0.83; 95% CI, 0.80 to 0.87).

Consistent with previous studies, mental health symptom severity was associated with increased odds of mental health-related service contacts.^1,5,7,8^ Of note, perception of a need for professional help for emotional and/or behavioural problems was the strongest predictor of service contact, independent of mental health symptom severity. Our results are in line with Canadian,^43,44^ European^45,46^ and Australian^47,48^ findings indicating a self-reported perceived need for mental health treatment is an important determinant of mental health service utilization. Negligible attenuation of the magnitude of the association of perceived need and mental health service contact after accounting for social and economic variables suggests there are other factors at play influencing parent identification of the need for help. Large differences in levels of perceived need were evident across non-migrant and migrant groups, highlighting the importance of adjusting for these in our analysis. Evidence suggests mental health literacy and social stigma are important factors giving rise to these group differences.^45,47^

Schools were the most frequented setting accessed by children and youth, as seen in previous literature.^1,7,8,31,32^ This highlights the importance of schools in addressing mental health concerns but also in acting as the first point of contact in a potential pathway of referrals, all of which are vital for early identification and intervention.

Interestingly, disparities in access across immigrant and refugee subgroups were found to be comparable in magnitude, contrary to prior evidence demonstrating overutilization among refugees, relative to immigrants.^6,21^ This could be because previous reports exclusively relied on physician-based administrative records (i.e., only those children and youth who have received mental healthcare from a physician), thereby capturing a limited proportion of children and youth and an even smaller number of refugee children and youth. The present study included a comprehensive range of settings and sectors where mental healthcare is delivered and thus included a broader cross-section of children and youth receiving mental health-related services, in addition to capturing adolescents in need who did not receive help. This is particularly important in the context of minority mental healthcare services research, given that these population subgroups have been shown to be consistently underserved.^49^ We found that a general population sample screened for perceived need and mental health service use are exhibiting a high need for professional help, yet underutilization of services. This treatment gap prompts the need for routine screening of treatable mental health conditions to identify and target high-risk adolescents.

This is the first study to our knowledge that has taken a comprehensive approach to examining disparities in mental healthcare across migrant subgroups, race and ethnicity, while accounting for between-group differences in mental health symptom severity and perceptions of the need for professional help. However, it is not without limitations. Generalizability of the findings outside of Ontario must be made with caution due to provincial and international variations in immigration policies, and healthcare and education systems. Another limitation lies in the reporting of outcomes as the results of this study were based on parent reports which may not accurately capture children's and youth's perceptions. Additionally, our assessment should not be equated with the receipt of treatment. We undertook a broad approach to capture any service contact for mental health concerns, as such, we are unable to characterize the nature of those contacts in relation to assessment, treatment referral, and intervention. The sampling strategy and eligibility criteria of the OCHS resulted in an absence of certain migrant groups such as non-English/French speaking children and youth, children without a CCTB tax file, as well as children living on reserves and institutions. Due to this, it is plausible that our results are an underestimation of the true magnitude of disparities in mental healthcare access. Given heterogeneity *within* migrant subgroups, future studies that have adequate sample coverage should take a more refined approach to examine within-group differences by migrant class (i.e., economic, family reunification), recency of arrival in Canada and more homogenous racial and ethnic subgroups.

Insights from this work could be used to implement programs and policies designed to reduce disparities in mental healthcare and promote mental health. To respond to the increasing diversity of immigrant, refugee and racial and ethnic minoritized populations in Canada, approaches to improve uptake and overcome barriers are needed, such as culturally tailored mental health training for service providers and community-based programs to improve mental health literacy. Equitable access to care requires investments in approaches that facilitate early identification and access to interventions across sectors and settings, as well as targeted outreach for immigrant, refugee and racial and ethnic minoritized children and youth.

## Supplemental Material

sj-docx-1-cpa-10.1177_07067437221144630 - Supplemental material for Social Disparities in Mental Health Service Use Among Children and Youth in Ontario: Evidence From a General, Population-Based Survey Click here for additional data file. Supplemental material, sj-docx-1-cpa-10.1177_07067437221144630 for Social Disparities in Mental Health Service Use Among Children and Youth in Ontario: Evidence From a General, Population-Based Survey by Mahdis Kamali, Jordan Edwards and Laura N. Anderson, Eric Duku, Katholiki Georgiades in The Canadian Journal of Psychiatry
