# Supplementary material for: Social Disparities in Mental Health Service Use Among Children and Youth in Ontario: Evidence From a General, Population-Based Survey
Source: Can J Psychiatry. 2022 Dec 12;68(8):596–604. doi: 10.1177/07067437221144630 (PMC10411367; doi:10.1177/07067437221144630)
Supplement: sj-docx-1-cpa-10.1177_07067437221144630 - Supplemental material for Social Disparities in Mental Health Service Use Among Children and Youth in Ontario: Evidence From a General, Population-Based Survey [file sj-docx-1-cpa-10.1177_07067437221144630.docx]

**Supplementary File 1:** Variable definitions and coding

| **Data Dictionary** | |
| --- | --- |
| **Variable** | **Definition and Coding** |
| **Demographics** | |
| **Age** | Parent was asked the year the child was born. Coded in years. |
| **Sex** | Male= 0 (Reference)  Female= 1 |
| **Race/Ethnicity** | Parents selected one or more categories that best described their race and ethnicity:  White= 0 (Reference)  Asian (East Asian, Southeast Asian, South Asian)  West Asian (West Asian, Arab)  Black (Black African, Black Caribbean, or Black Canadian or American)  Other (Aboriginal, First Nations, Metis, Inuit, Latin American, Other) |
| **Family Structure** | Parent was asked who the child primarily lives with:  2 biological parents in the home= 0 (Reference)  2 parents (either 1 biological and 1 step-parent, adoptive parent, or foster parent; or 2 step-parents/adoptive/foster),  Single parent (biological mother only, biological father only, non-biological parent, other female or male parent) |
| **Household Income** | Household income below the low-income measure (based on the 2013 before-tax cutoffs derived from Statistics Canada)  Above low-income measure = 0 (Reference)  Below low-income measure = 1 |
| **Social assistance** | Parent report on whether provincial or municipal social assistance or welfare was a source of total household income in the last 12 months  No = 0 (Reference)  Yes = 1 |
| **Parental Education** | Parent and partner’s highest level of education. This variable is a measure of the highest level of education between the parent and partner.  Bachelors or higher = 0 (Reference)  Diploma, trade certificate = 1  Highschool or less= 2 |
| **Parental Employment** | PMK was asked of their current employment.  Full-time = 0 (Reference)  Part-time  Looking  Unemployed |
| **Rurality** | Household urbanicity derived from Census 2011 and classified by Statistics Canada based on postal code.  Rural= 0 (Reference)  Urban (small-med urban, large urban) = 1 |
| **Migrant Status** | Based on parent and partner report on their country of birth and migration status.  Non-immigrant (both parents born in Canada) = 0 (Reference)  Immigrant (one parent arrived in Canada as landed immigrant)  Refugee (one parent arrived in Canada as refugee) |
| **Discrimination** | Parents were asked whether they have felt that they have experienced discrimination or been treated unfairly by others in Canada because of their ethnicity, culture, race, skin colour, language, accent, or religion  No = 0 (Reference)  Yes = 1 |
| **Need** | |
| **Internalizing and Externalizing Symptoms** | Measured through dimensional ratings from the 2014 Ontario Child Health Study Emotional Behavioural Scales (OCHS- EBS)(38).  Total, summative scores were used with a higher score indicating greater symptom severity. |
| **Perception of Need** | Parents were asked whether they perceived a need for professional help with emotional or behavioural problems for their child  No = 0 (Reference)  Yes = 1 |
| **Outcome Service Contact** | |
| **Service Contact** | Parent report on whether child has made contact for mental health-related concerns in the past six months (by provider type and service setting)  No = 0 (Reference)  Yes = 1 (spoke to general health care providers (family doctor, pediatrician, other health professional), mental health providers (psychiatrist, psychologist, social worker, other type of counsellor), school guidance counsellor or teacher/other adult at school, a combination of complementary/alternative medicine providers (religious or spiritual leader, alternative healers such as a naturopath or herbalist) or phone helpline or crisis hotline, gone to walk-in clinic, urgent care clinic or hospital, school-based setting, or an agency that provides mental health or addiction services for mental health-related concerns) |
